# Correlations of religious beliefs with anxiety and depression of Chinese adolescents

**DOI:** 10.3389/fpsyt.2024.1354922

**Published:** 2024-03-01

**Authors:** Lejun Li, Xiliang Liu, Pingping Wang, Miao Qu, Meihong Xiu

**Affiliations:** ^1^ Department of Neurology, Suzhou TCM Hospital Affiliated to Nanjing University of Chinese Medicine, Suzhou, China; ^2^ Qingdao Mental Health Center, Qingdao, China; ^3^ Neurology Department, Xuan Wu Hospital of Capital Medical University, Beijing, China; ^4^ Peking University Huilongguan Clinical Medical School, Beijing Huilongguan Hospital, Beijing, China

**Keywords:** adolescents, religious belief, depression, anxiety, association

## Abstract

**Objective:**

This study was designed to investigate the prevalence of religious belief and its relationship with psychiatric symptoms among Chinese adolescents.

**Methods:**

This study recruited 11,603 adolescents in Grades 7-9 from March 21 to 31, 2020 in five cities in China. The religious beliefs of adolescents were collected by asking whether they held religious beliefs and what type of religious beliefs they held. The Patient Health Questionnaire-9 (PHQ-9) and the Generalized Anxiety Disorder-7 Scale (GAD-7) were used to assess depressive and anxiety symptoms in all adolescents. Demographics, religious beliefs, and mental health status were collected through the professional version of Wenjuanxing.

**Results:**

Of 11,069 valid questionnaires collected, 847 (7.7%) reported holding religious beliefs. Adolescents with religious beliefs showed significantly more severe symptoms of depression and anxiety compared to those without religious beliefs (both p<0.05). Logistic regression analysis revealed that religious belief was a risk factor for symptoms of depression (OR = 1.37, 95%CI: 1.16-1.61, p < 0.001) and anxiety (OR = 1.49, 95%CI: 1.23-1.79, p < 0.001) after controlling age, gender, and parental marital status.

**Conclusions:**

Our findings suggest that religiousness in adolescents was associated with a higher likelihood of depression/more intense depressive symptoms. In addition, religious Chinese adolescents should be provided with more resources to help them cope with mental health concerns.

## 1 Introduction

Mental health problems affect 10-20% of adolescents worldwide (1). Studies in adolescents have shown that their depressive symptoms are associated with poor health status, new medical illnesses and disability. Common pathways related to mental health and behavioral problems in adolescents include genetic vulnerabilities and early neuroendocrine changes, personality traits, violence, using substances, experiencing somatic symptoms, poor school and family support, and stressful life events (2, 3).

Adolescence is a critical period for human cognitive and behavioral development (4). In addition, during adolescence, individuals develop the capacity to understand and internalize religious beliefs (5). Adolescents have their views on religion and its accompanying beliefs and practices, and some of them question or reject some aspects of religious ideas taught to them at their age (6). Communication with others and receiving religious education are critical to developing religious beliefs, considering that they promote awareness about religious beliefs and practices and how these affect individuals and the family. Religiosity and spirituality include the following domains: beliefs, attitudes, and behaviors (7, 8). The effects of religious beliefs on mental health have been investigated in previous studies (9). Robust evidence suggests that religious beliefs may be associated with the severity of depressive symptoms in adults (10). In particular, previous meta-analyses of randomized controlled trials examining the impact of religious intervention have reported positive effects on mental health, such as significant reductions in depressive symptoms (11, 12). On the other hand, several studies have shown that religious beliefs or behaviors have a positive impact on alcohol users, drug users, and individuals with suicidal ideation (13, 14).

However, findings on the association of religious involvement with depressive symptoms were inconsistent (15, 16). A few studies have shown that religious belief was positively associated with longevity (17, 18) and negatively correlated with depressive symptoms (19). A meta-analysis of the relationship between religious/spiritual beliefs and depression or depressive symptoms in 147 studies (N = 98,975) reported a negative correlation between religious belief and depressive symptoms (10). Especially after recent severe stressful life events, religious beliefs had a stronger protective effect on depressive symptoms.

Nonetheless, it remains unknown whether religious beliefs during adolescence have a positive impact on the mental health of adolescents. A study by Estrada reported that religious education in school settings played an important role in improving adolescent mental health (4). It is worth noting that in China, only a small percentage of people have religious beliefs. Most of them develop religious beliefs under the influence of their close family members and may adopt their parents’ religious beliefs and practices (20). Currently, the rate of religiosity among Chinese adolescents and its impact on mental health has not been studied. Hence, in this study, we aimed to investigate the relationship between religious belief and depressive symptoms in adolescents (Grade 7, 8, and 9).

## 2 Methods

### 2.1 Subjects and design

This cross-sectional study was conducted from March 20 to 31, 2020 in five middle schools located in five cities, including Shanxi, Shandong, Henan, Fujian and Liaoning, respectively. Students aged 12-18 years in Grades 7, 8 and 9 were recruited and an online questionnaire was used to collect data. Prior to the survey, parental permission was obtained, and 50 teachers involved in data collection received online training in the use of the “SurveyStar” network platform to ensure the accuracy and reliability of the survey. All trained teachers then posted the survey link to participants after training and instructed them to complete and submit the survey. As in previous studies (21–24), We set criteria for invalid data. All adolescents voluntarily participated in this study.

We adopted the method of whole sampling, and all the students of the schools were surveyed according to our protocol. The criteria for invalid questionnaires were as follows: 1) the completion rate of the paper questionnaire was less than 90%, 2) unreasonable or illogical data were found, 3) important data such as gender and age were not filled in, and 4) the researcher confirmed that the student did not fill in questionnaire carefully. Questionnaires with any of these criteria were considered invalid. Finally, 11,069 adolescents with valid data were recruited in this study.

This study protocol was approved by the Ethics in Human Research Committee of the Third Affiliated Hospital of Beijing University of Chinese Medicine. Online informed consent was obtained from all students or their guardians.

### 2.2 Assessments

A structured self-assessment questionnaire was designed to collect demographic data and symptoms of depression and anxiety. In this online survey, adolescents were asked whether they held religious beliefs and what type of religious beliefs they believed. Those who answered “yes” were recorded as religious and others were recorded as non-religious. We found that the major religious beliefs included Buddhism, Christianity, Islam and others in this study.

Depressive symptoms of adolescents were assessed using a Chinese version of the Patient Health Questionnaire (PHQ-9) (25, 26), which consists of nine items. The scores range from “nearly every day” (3 points) to “not at all” (0 points). The Cronbach alpha in this study was 0.851. In this study, the threshold for depressive symptoms was set at a score of 5. Anxiety symptoms were assessed by the Chinese version of the Generalized Anxiety Disorder Scale (GAD-7) (27, 28). Scores 0, 1, 2 and 3 usually represent the categories of “not at all”, “a few days”, “more than half a day” and “almost every day” to calculate GAD-7 scores. The Cronbach alpha in this study was 0.837. In this study, the threshold for anxiety was set at >5.

### 2.3 Statistical analyses

All analyses were performed in SPSS, version 20.0. Shapiro-Wilk test and Q-Q plot were used to test normality for continuous variables. The continuous variables of non-normal distribution were described using the median and interquartile ranges (IQRs).

Adolescents were categorized into a religious group and a non-religious group, according to whether they held religious beliefs. Chi-square tests, the non-parametric Mann-Whitney U test, and the Kruskal-Wallis test when comparing more than 2 groups were performed as appropriate to compare data between groups. Spearman’s correlation was performed to assess the association between religious beliefs and symptoms. To better elucidate the impact of religious belief on depressive and anxiety symptoms, logistic regression analysis was conducted to measure the ORs and 95% CI between religious belief and the outcomes.

To assess how large the differences between groups, the effect size of Cohen’s *d* was calculated for the comparisons (29). The effect size was interpreted in light of Cohen’s (1992) recommendations: an effect size <0.3 was considered small, an effect size of 0.5 was considered medium, and an effect size above 0.8 was considered large.

All p values were 2-tailed and the significance level was <0.05.

## 3 Results

Of the initial 11,603 adolescents, 11,069 questionnaires (95.4%) were valid after excluding invalid questionnaires. Comparison of the demographic and psychological characteristics between the two groups is shown in **Table 1**. There were 5592 boys (50.5%) and 5477 girls (49.5%). The average age of all students was 14.3 years. The rate of depressive symptoms in adolescents was 37.5% (4,156/11,069) and the rate of anxiety symptoms was 23.4% (2,590/11,069). Depressive symptoms were significantly associated with sex (p<0.001). Girls had higher levels of depressive and anxiety symptoms than boys.

**Table 1:** Demographic and psychological characteristics of adolescents.

	Total adolescents n=11,069	Religious group n=847	Non-religious group n=10,222	*p* value
Demographic Characteristics				
Age, mean ± SD	14.3 ± 1.1	14.3 ± 1.1	14.5 ± 1.0	0.001
Boys, n (%)	5592 (50.5)	432 (51.0)	5160 (50.5)	0.77
Marital status of parents				0.13
Married	10082 (91.1)	775 (91.5)	9307 (91.0)	
Divorced	487 (4.4)	26 (3.1)	461 (4.5)	
Remarried	278 (2.5)	27 (3.2)	251 (2.5)	
Single	222 (2.0)	19 (2.2)	203 (2.0)	
Psychological Characteristics				
PHQ-9, median (IQR)	3 (0, 7)	4 (1, 9)	3 (0, 7)	<.0001
PHQ-9, mean ± SD	4.5 ± 5.3	5.4 ± 6.0	4.4 ± 5.2	<.0001
With symptoms, n (%)	4156 (37.5)	371 (43.8)	3785 (37.0)	<.0001
GAD-7, median (IQR)	1 (0, 4)	1 (0, 6)	1 (0, 4)	<.0001
GAD-7, mean ± SD	2.8 ± 4.2	3.5 ± 4.8	2.7 ± 4.1	<.0001
With symptoms, n (%)	2590 (23.4)	261 (30.8)	2329 (22.8)	<.0001

PHQ-9, 9-item Patient Health Questionnaire; GAD-7, 7-item Generalized Anxiety Disorder.

### 3.1 Prevalence of religious belief and its association with mental health status

The rate of religious beliefs among adolescents was 7.7% (847/11,069). Of all religious believers, 682 (80.5%) were BUDDHIST, 100 (11.8%) were CHRISTIAN, 22 (2.6%) were MUSLIM and 43 (5.1%) were affiliated with other religions. The average age of the religious group was slightly lower than that of the non-religious group (p= 0.001).

The rate of depressive symptoms was different between the adolescents with religious beliefs and non-religious beliefs (37.0% vs 43.8%; *X^2 =^* 15.3, p< 0.001). There was also a significant difference in the rate of anxiety between the adolescents with religious beliefs and non-religious beliefs (22.8% vs 30.8%; *X^2 =^* 28.1, p< 0.001).

Further analysis found that compared to those non-religious adolescents, adolescents with religious beliefs had more severe depressive symptoms (Z= 4.8, p< 0.001) (effect size= 0.178) and anxiety symptoms (Z= 5.0, p< 0.001) (effect size= 0.179). The Cohen’s *d* showed a small effect. After controlling for age, sex and parental marital status, the differences between the two groups remained significant (all p< 0.01). There were no significant differences in depressive (p= 0.07) and anxiety symptoms (p= 0.06) among adolescents in the four religious categories (**Table 2**).

**Table 2:** Depression and anxiety between the different religious groups.

	Buddhist (N=682)	Christian (N=100)	Muslim (N=22)	Other (N=43)	Statistic (p)
Depression	5.2 ± 5.8	5.5 ± 6.3	7.5 ± 7.3	8.2 ± 7.8	7.0 (0.07)
Anxiety	3.3 ± 4.6	3.2 ± 4.6	5.6 ± 6.1	6.4 ± 7.4	7.3 (0.06)

### 3.2 Factors associated with depressive and anxiety symptoms

Multiple regression and logistic regression analyses were carried out to confirm the association between religious beliefs and symptoms. In these models, religious belief, age, parental marital status and sex were used as independent variables, and symptoms were added as dependent variables. We found that religious belief, older age, and girls were associated with depressive symptoms (p< 0.05, *R^2 =^* 0.41) (**Table 3**). In addition, religious belief, girls, and older age were also found to be associated with anxiety symptoms (p< 0.01, *R^2 =^* 0.34). The coeffcients of the variables are shown in **Table 4**. Logistic regression analysis found that religious belief was a risk factor for depressive symptoms (OR = 1.37, 95%CI: 1.16-1.61, p < 0.001) and anxiety symptoms (OR = 1.49, 95%CI: 1.23-1.79, p < 0.001) (**Tables 3**, **4**).

**Table 3:** Results of the multivariate regression models predicting depression.

Variables	Linear regression analysis β (95%CI)	t (p)	Logistic regression analysis OR (95%CI)	*Wals X* ^2^ (p)
Age	0.21 (0.14, 0.28)	6.0 (<0.001)	0.90 (0.86, 0.94)	26.7 (<0.001)
Sex	0.65 (0.49, 0.80)	8.3 (<0.001)	1.4 (1.28, 1.53)	54.8 (<0.001)
Marital status of parents	0.13 (-0.01, 0.27)	1.8 (0.08)	1.0 (0.87,1.20)	0.09 (0.77)
Religious belief	0.76 (0.47, 1.04)	5.3 (<0.001)	1.37 (1.16, 1.61)	13.3 (<0.001)

**Table 4:** Results of the multivariate regression models predicting anxiety.

Variables	Linear regression analysis β (95%CI)	t (p)	Logistic regression analysis OR (95%CI)	*Wals X* ^2^ (p)
Age	0.12 (0.07, 0.18)	4.2 (<0.001)	0.92 (0.88, 0.96)	13.8 (<0.001)
Sex	0.52 (0.39, 0.65)	8.0 (<0.001)	1.43 (1.29, 1.59)	45.3 (<0.001)
Marital status of parents	0.12 (-0.01, 0.23)	1.9 (0.05)	1.02 (0.85,1.22)	0.04 (0.84)
Religious belief	0.62 (0.38, 0.85)	5.2 (<0.001)	1.49 (1.23, 1.79)	17.7 (<0.001)

## 4 Discussion

There were two major findings in our study. 1) The frequency of religious belief among adolescents (grades 7-9) was 7.7% (847/11,069). 2) Adolescents with religious beliefs had more severe symptoms of anxiety and depression.

To our knowledge, it was the first study to investigate the prevalence of religious beliefs among Chinese adolescents. We found that the rate was only 7.7% among adolescents. This was similar to another study in rural China, which reported 7.8% of the general population in Hunan and Shandong provinces (30). The prevalence of religiosity among adolescents in different provinces of China varies due to its high prevalence in certain provinces. In this study, the adolescents were recruited from cities in China where religion is less prevalent, making them more representative of the general population.

This study found that religious adolescents had more severe depressive and anxiety symptoms, which is consistent with several prior studies in the United States, Canada and Korea (31, 32). It should be noted that there were inconsistencies in the association between religious beliefs and depression among individuals of different ages and sex, from a wide range of cultural backgrounds, and with different religious orientations in previous studies (10, 11, 33, 34). Some studies have reported inverse relationships between religious beliefs and psychiatric symptoms (17, 18). For example, a multi-generational 10-year prospective study of young adults in the United States found that religious beliefs showed a protective effect on depressive symptoms, particularly relapse (35, 36). We speculate the following possible explanations for this link between religious belief and symptoms. Recent research suggests that two opposite epidemiological forces of religion lead to associations between religiosity and mental health status (37). Some adolescents maintain religious beliefs intrinsically, primarily because they have an intrinsic rest in their faith, and due to internalization over time within the family environment (38). Family structure and attachment play an important role in influencing how religious beliefs and behaviors are transmitted to adolescents. It has been reported that children raised by their parents with close family relationships may follow their parents’ religious beliefs [20]. Although previous studies have also reported that peers influence adolescents’ religiosity because peers who share the same religious beliefs are likely to foster more close relationships, and tend to have more friends who share the same religious beliefs. In China, very few of them acquire religious beliefs from their peers because it is known that China has the largest irreligious population in the world, especially among adolescents (39). Thus, we speculate that religious and spiritual beliefs were mainly transmitted from family members.

Indeed, from a biological perspective, Kendler and his colleagues conducted a genetic epidemiological study of adult female twins with an average age of 30.1 years (n = 2,163) and found that broad heritability accounted for 29% of the variance in religious beliefs (40). On the other hand, severe diseases and crises also promote the prevalence of religious beliefs. It is well known that disease stimulates religious seeking, often referred to as the “religious coping mobilization effect”. Hence, depressive symptoms may motivate adolescents to seek and maintain religious beliefs. Nonetheless, it should be noted that religious beliefs and behaviors have been recognized to be deviant in China, despite that freedom of religion is protected by the constitution (41). Twenty years ago, some religious activities in China were controlled by the government. Therefore, this situation made religious people more vulnerable to mental illness. Adolescents with depressive symptoms who maintain religious beliefs may be treated differently from non-religious adolescents, but they are more likely to be stigmatized by their religious beliefs. Adolescents with religious beliefs may experience negative emotions and anxiety, which increase the risk of anxiety and depression. In short, all these reasons can explain why religious adolescents are more prone to experience symptoms of anxiety and depression.

Our study has several limitations. First, some protective and risk factors for depression and anxiety, such as trauma, family dynamics, and social support, were not included in the present study. In particular, social support, including social activities or contact with fellow churchgoers and friends, as a general construct, was not included in the analyses. Further studies should include more relevant factors to investigate the complex relationship between religious beliefs and mental health status. Second, a methodological limitation is that we used a short two-item interview to assess religious beliefs. We did not comprehensively measure other aspects of religiosity, such as religious participation, spiritual help-seeking, religious coping, or satisfaction with religious beliefs. Had we used more comprehensive measures of religiosity, we could have obtained a more nuanced picture of the complex relationships between religion and mental health. Third, due to the lack of religious knowledge among the Chinese public, adolescents with religious beliefs may be treated differently from non-religious adolescents. Thus, it is common in survey respondents to not disclose/hide their religious beliefs, which is another source of bias in the data that deserves consideration in interpreting the findings of this study. Fourth, we did not collect data on adolescents’ history of psychiatric illness or family history of psychiatric disorders.

In conclusion, our findings show that adolescents with religious beliefs had more severe symptoms of anxiety and depression compared to those without religious beliefs. Our findings suggest that religious Chinese adolescents should be provided with more resources to help them cope with mental health concerns. However, this was a cross-sectional study. Further prospective studies using more sophisticated and sensitive measures of religiosity are warranted to verify these findings and elucidate underlying mechanisms.

## Data availability statement

The raw data supporting the conclusions of this article will be made available by the authors, without undue reservation.

## Ethics statement

This study protocol was approved by the Ethics in Human Research Committee of the Third Affiliated Hospital of Beijing University of Chinese Medicine, and each student provided online informed consent. The studies were conducted in accordance with the local legislation and institutional requirements. The participants provided their written informed consent to participate in this study.

## Author contributions

LL: Writing – review & editing, Data curation. XL: Writing – review & editing, Data curation. MX: Investigation, Writing – original draft. PW: Conceptualization, Software, Writing – original draft. MQ: Writing – original draft, Writing – review & editing.
